# Trends in Trans Fatty Acids Reformulations of US Supermarket and Brand-Name Foods From 2007 Through 2011

**DOI:** 10.5888/pcd10.120198

**Published:** 2013-05-23

**Authors:** Fadar O. Otite, Michael F. Jacobson, Aspan Dahmubed, Dariush Mozaffarian

**Affiliations:** Author Affiliations: Fadar O. Otite, Harvard School of Public Health, Boston, Massachusetts; Michael F. Jacobson, Aspan Dahmubed, Center for Science in the Public Interest, Washington, DC.

## Abstract

**Introduction:**

Although some US food manufacturers have reduced trans fatty acids (TFA) in their products, it is unknown how much TFA is being reduced, whether pace of reformulation has changed over time, or whether reformulations vary by food type or manufacturer.

**Methods:**

In 2007, we identified 360 brand-name products in major US supermarkets that contained 0.5 g TFA or more per serving. In 2008, 2010, and 2011, product labels were re-examined to determine TFA content; ingredients lists were also examined in 2011 for partially hydrogenated vegetable oils (PHVO). We assessed changes in TFA content among the 270 products sold in all years between 2007 and 2011 and conducted sensitivity analyses on the 90 products discontinued after 2007.

**Results:**

By 2011, 178 (66%) of the 270 products had reduced TFA content. Most reformulated products (146 of 178, 82%) reduced TFA to less than 0.5 g per serving, although half of these 146 still contained PHVO. Among all 270 products, mean TFA content decreased 49% between 2007 and 2011, from 1.9 to 0.9 g per serving. Yet, mean TFA reduction slowed over time, from 30.3% (2007–2008) to 12.1% (2008–2010) to 3.4% (2010–2011) (*P* value for trend < .001). This slowing pace was due to both fewer reformulations among TFA-containing products at start of each period and smaller TFA reductions among reformulated products. Reformulations also varied substantially by both food category and manufacturer, with some eliminating or nearly eliminating TFA and others showing no significant changes. Sensitivity analyses were similar to main findings.

**Conclusions:**

Some US products and food manufacturers have made progress in reducing TFA, but substantial variation exists by food type and by parent company, and overall progress has significantly slowed over time. Because TFA consumption is harmful even at low levels, our results emphasize the need for continued efforts toward reformulating or discontinuing foods to eliminate PHVO.

## Introduction

Trans fatty acids (TFA) are monounsaturated or polyunsaturated fats with at least 1 *trans* double bond, rather than the *cis* double bond normally synthesized by mammals and most plants (1). Small amounts of TFA occur naturally in meats and dairy products, but most TFA in modern diets are the result of industrially produced partially hydrogenated vegetable oils (PHVO). Partial hydrogenation converts vegetable oils to semisolid fats that have attractive commercial properties for cooking, baking, and frying. The use of PHVO — and corresponding TFA content of foods — increased substantially during the 20th century. In recent years, evidence for cardiovascular and metabolic harms of TFA (1–4) in conjunction with federal nutrition labeling and regional limitations on TFA (5–7) have spurred some US companies to reformulate their products to reduce TFA. Estimates from National Health and Nutrition Examination Survey (NHANES) data suggest that mean US consumption of industrially produced TFA (IP-TFA) decreased between 2003 and 2006 (8) and, on the basis of blood TFA levels, that average US exposure declined by approximately 50% between 2000 and 2009 (9). We previously reported on 83 brand-name foods from US supermarkets and restaurants that were all reformulated to reduce TFA from 1993–2006 through 2008–2009 (10). Among these reformulated products, average TFA content was reduced 92%; we found a mean reduction of 1.8 g per serving (standard deviation [SD], 2.3 g/serving) in supermarket foods and 3.2 g per serving (SD, 1.5 g/serving) in restaurant foods, generally without increases in saturated fatty acids (SFA). However, we did not evaluate the denominator (ie, the products that were not reformulated).

Overall, this evidence suggests that PHVO use in the United States is decreasing. However, the pace at which such reformulations are occurring remains unclear, and it may also vary over time, by type of product, and between companies. We investigated the trends in reformulation of major brand-name TFA-containing products in the United States from 2007 through 2011, including whether the pace of reformulations increased, decreased, or remained similar over time and how reformulation efforts varied by type of food product and company.

## Methods

### Identification of TFA-containing products in 2007

In 2007, Center for Science in the Public Interest staff visited large US supermarkets to identify major brand-name products, including major supermarket brands, that contained TFA. Stores included Safeway in Washington, DC, Giant Foods in Washington, DC, and Walmart in Maryland, based on their extensive stocking of major national products and their own store brands. In each store, the Nutrition Facts panels of all products in 16 categories of foods likely to contain IP-TFA (breads; breakfasts; cakes and pastries; cookies, biscuits, and bars; crackers; doughnuts; French fries or other potatoes; ice creams; margarines and spreads; meats and seafood; muffins; pasta; pies; pizzas; popcorns; and rolls) were inspected to identify foods labeled as containing 0.5 g or more per serving TFA. Given the breadth and numbers of products evaluated in 2007 and focus on potential reformulations, ingredient lists were not separately searched in 2007 to identify foods that might contain less than 0.5 g IP-TFA per serving (ie, based only on the presence of PHVO in the ingredient list). For each identified product containing 0.5 g or more per serving TFA in 2007, data were collected on TFA content, product type, name, brand, and parent company.

### Assessment of product formulations by 2011

All identified products containing 0.5 g or more per serving TFA in 2007 were re-examined in 2008, 2010, and 2011 (data were not collected in 2009). In each evaluation year, TFA content was re-evaluated and recorded; SFA content was also recorded in 2010 and 2011. Because the US Food and Drug Administration allows foods with less than 0.5 g per serving TFA to list “0 g” on the Nutrition Facts panel while also containing PHVO (11), detailed ingredient lists of each product were also evaluated in 2011 for the presence of PHVO. Foods listing 0 g per serving TFA in the Nutrition Facts and having no PHVO in the ingredient list were considered to contain 0 g TFA. Products listing 0 g per serving TFA but still containing PHVO were considered to contain 0.25 g per serving TFA, selected as the median potential content between 0 and 0.5g. Use of a higher assumed content of 0.4 g per serving for such products had little effect on results. Our direct gas chromatographic analysis of 3 products listing 0 g TFA but containing PHVO demonstrated a mean TFA content of 0.3 g per serving. Foods reformulated in 2008 or 2010 that listed 0 g TFA were considered to contain 0.25 g per serving in those years if they contained PHVO in 2011. For any products not in stores during follow-up visits in 2011, up-to-date product information (including that the products were no longer marketed) was obtained from food manufacturers by direct telephone calls (n = 25 products) or their online nutrition information (n = 78 products).

### Statistical analysis

We assessed quantitative changes in TFA content of products during each successive pair of years of data collection (periods: 2007–2008, 2008–2010, and 2010–2011) and overall (2007–2011). Mean levels among all products were compared by using paired *t* tests, and mean levels among subsets of products (eg, only those reducing TFA, only those not reducing TFA) by using unpaired *t* tests. We similarly assessed quantitative changes in SFA and a combination of TFA and SFA during 2010 and 2011, the years with available SFA data. Changes were assessed as changes in both grams per serving and percentage of TFA content. Potential differences in pace of reformulation over time were evaluated by using linear regression with TFA change as the dependent variable and years of change as the independent variable, evaluated continuously, with significance of differences in changes over time assessed by using the Wald test. We also quantified changes according to categories of food product and by company.

Of 360 identified major brand-name products containing 0.5 g or more per serving TFA in 2007, 90 were discontinued before 2011 (confirmed by direct contact with manufacturers). Because products may be discontinued for many reasons other than TFA content, our primary analyses focused on the 270 products sold in all years from 2007 through 2011. In sensitivity analyses, we included the 90 products that were discontinued over time, using actual TFA contents until discontinuation and then, following discontinuation, treating these products as still being in the market but containing 0 g TFA, to test the potentially largest trends in reformulations over time (ie, making the assumption that a discontinued product represents a “reformulated” product free of TFA). Because we investigated values over multiple periods, significance was defined as a 2-tailed α of .01. Analyses were performed using Stata 11 (StataCorp, LP, College Station, Texas). 

## Results

The 270 US brand-name TFA-containing foods in our primary analysis comprised an extensive range of products, representing 48 major brands or manufacturers including General Mills, Kellogg Company, Heinz, ConAgra, Safeway, Giant, Campbell Soup, Sara Lee, and Walmart (Appendix A). By 2011, 178 products (66%) had been reformulated to reduce TFA (Table 1), with a mean absolute decline in TFA among these 178 products of 1.5 (SD, 1.3) g per serving and a mean percentage decline in TFA of 78% (SD, 26) (Table 2). Among these 178 products, 146 (82%) listed 0 g TFA on their Nutrition Facts panels in 2011, but only half of these (n = 73) had completely eliminated IP-TFA (no PHVO in the ingredients list). Half (n = 73) still contained PHVO and were assumed to contain 0.25 g TFA in all analyses. Thirteen of the original 270 products increased their TFA content from 2007 through 2011, with an average increase of 0.7 (SD, 0.2) g per serving (see supplementary Table 1, Appendix B).

**Table 1:** Trans-fatty Acid (TFA) Reformulations From 2007 Through 2011 of 270 Major US Brand-Name Food Products That Contained ≥0.5 g/serving TFA in 2007 and Still Marketed in 2011

Reformulation	Year^a^			
	2007	2008	2010	2011
**All products**				
No. (%) of products containing any TFA^b^	270 (100)	231 (86)	203 (75)	197 (72)
Mean (SD) TFA content, g/serving^c^	1.9 (1.3)^*^	1.3 (1.3)†	1.0 (1.3)‡	0.9 (1.2)§
Median (IQR) TFA content, g/serving^c^	1.5 (1.0–2.5)	1.0 (0.25–2.0)	0.25 (0.25–1.5)	0.25 (0.0–1.5)
Cumulative no. (%) of products reformulated to reduce TFA	NA	123 (46)	162 (60)	178 (66)
Cumulative no. (%) of products reformulated to reduce TFA to <0.5 g/serving^b^	NA	97 (36)	137 (51)	146 (54)
Cumulative no. (%) of products reformulated to fully eliminate industrial TFA^b^	NA	39 (14)	67 (25)	73 (27)
Cumulative no. (%) of products reformulated to increase TFA^b^^,^ d	NA	17 (6)	16 (6)	13 (5)
**Products containing at least 0.5 g/serving TFA**				
No. of products containing at least 0.5 g/serving TFA	270	173	133	124
Mean (SD) TFA content, g/serving^e^	1.9 (1.3)^*^	1.9 (1.3)^*^	1.9 (1.3)^*^	1.9 (1.3)^*^
Median (IQR) TFA content, g/serving^e^	1.5 (1.0–2.5)	1.5 (1.0–2.5)	1.5 (1.0–2.5)	1.5 (1.0–2.0)

Abbreviations: SD, standard deviation; IQR, interquartile range; NA, not applicable.

a Data were not collected in 2009. Values with different symbols (*, †, ‡, §) are significantly different from each other (*P* < .01); values with identical symbols are not significantly different from each other.

b Products listing 0 g TFA but still containing partially hydrogenated oils in the ingredients list were considered to still contain 0.25 g per serving of TFA and were not included as eliminating industrial TFA.

c Values are among all 270 products. Large SD may represent heterogeneity in the TFA content of sampled products.

d Some products that were initially reformulated to increase their TFA later decreased their TFA content.

e Values are only among products that continued to contain at least 0.5 g per serving of TFA.

**Table 2:** Changes in TFA Content From 2007 Through 2011 of 270 Brand-Name US Food Products that Contained ≥0.5 g/serving TFA in 2007 and Still Marketed in 2011

Product	Overall (2007 to 2011)	Changes by Period^a^			*P* Value for Trend^c^
		2007 to 2008	2008 to 2010^b^	2010 to 2011	
**All products**					
No. of products	270	270	270	270	NA
Mean (SD) TFA change, g/serving^d^	−1.0 (1.3)	− 0.6 (1.2)^*^	−0.3 (0.9)†	−0.1 (0.4)‡	<.001
TFA % change, mean (SD)^d^^,^ e	−48.5 (50.0)	− 30.3 (48.4)^*^	−12.1 (65.9)†	−3.4 (79.7)‡	<.001
**Products that reduced TFA**					
No. of products containing TFA at the start of the period	270	270	231	203	NA
No. (%) of products reducing TFA	178 (66)	123 (40)	72 (31)	31 (15)	NA
No. (%) that reduced to <0.5 g/serving	146 (54)	97 (36)	49 (21)	16 (8)	NA
No. that reduced to 0 g/serving (%)^d^	73 (27)	39 (14)	28 (12)	6 (3)	NA
Mean (SD) TFA change, g/serving^d^	−1.5 (1.3)	−1.5 (1.3)^*^	−1.3 (1.2)^*^	−1.0 (0.7)^*^	<.001
Mean (SD) TFA % change^d^^,^ e	−78.4 (26.1)	−75.0 (26.8)^*^	−71.4 (30.7)^*^†	−61.3 (28.1)†	.03
**Products containing** ≥**0.5 g/serving TFA that reduced TFA**					
No. of products containing ≥0.5 g/serving TFA at the start of the period	270	270	173	133	NA
No. (%) of products reducing TFA	178 (66)	123 (40)	67 (39)	31(23)	NA
TFA change, g/serving, mean (SD)	−1.5 (1.3)	−1.5 (1.3)^*^	−1.4 (1.2)^*^	−1.0 (0.7)^*^	.10
Mean (SD) TFA % change^e^	−78.4 (26.1)	−75.0 (26.8)^*^	−69.2 (30.8)^*^	−61.3 (28.1)^*^	.02
**Products that increased TFA**					
No. of products	13	17	19	15	NA
Number increased from <0.5 g/serving		—	3	3	NA
Mean (SD) TFA change, g/serving^d^	0.7 (0.2)	0.7 (0.4)^*^	0.9 (0.5)^*^	0.7 (0.5)^*^	.86
Mean (SD) TFA % change^d^^,^ e	64.6 (59.2)	60.0 (48.7)^*^	123.9 (130.8)^*^	172.8 (223.6)^*^	.03

Abbreviations: NA, not applicable; SD, standard deviation.

a Values with different symbols (*, †, ‡) are significantly different from each other (*P* < .01) across periods; values with identical symbols are not significantly different from each other.

b A 2-year period; data were not collected in 2009.

c Determined from linear regression model with TFA change (either in g/serving or percentage) modeled as dependent variable and time (in years) modeled as a continuous variable.

d All products listing 0 g TFA but still containing partially hydrogenated oils in the ingredients list were considered to still contain 0.25 g per serving of TFA and were not included as eliminating industrial TFA.

e Mean gram per serving changes and mean percentage changes are summed at the individual product level and are thus not mathematically identical.

Among all 270 products, the mean TFA content decreased from 1.9 (SD, 1.3) g per serving in 2007 to 0.9 (SD, 1.2) g per serving in 2011 (*P* for change < .001), an overall decline of 49%. This decline was predominantly due to reformulations that reduced TFA to less than 0.5 g per serving. Among products that continued to contain 0.5 g or more per serving, average TFA content did not significantly change over time (1.9 [SD, 1.3] g/serving in 2007 vs 1.9 [SD, 1.3] g/serving in 2011).

**Figure 1:**
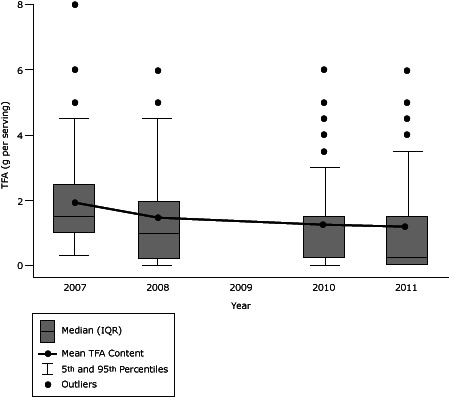
Trans-fatty acid (TFA) content from 2007 through 2011 of 270 brand-name US supermarket food products that contained TFA in 2007 and were still marketed in 2011. In 2010, the 25th percentile and median values were identical (0.25 g/serving). All *P* values < .01 comparing mean TFA content of all products in each year to mean TFA content in the following year of data collection. Products listing 0g TFA but still containing partially hydrogenated vegetable oils in the ingredients list were considered to contain 0.25 g/serving of TFA and were not included as eliminating industrial TFA. Data were not collected in 2009. Abbreviations: SE, standard error; IQR, interquartile range. **Table:** 

The pace of TFA reduction significantly decreased over time (Figure 1). The mean reduction among all products was 0.6 (SD, 1.2) g per serving (30% decline) from 2007 through 2008, 0.3 (SD, 0.9) g per serving (12% decline) from 2008 through 2010, and 0.1 (SD, 0.4) g per serving (3% decline) from 2010 through 2011 (*P* value for trend, < .001) (Table 2). This slowing in TFA reduction was due to fewer reformulations over time; restricting calculations to only those products that contained TFA at the start of each period, 40% were reformulated from 2007 through 2008, 31% from 2008 through 2010, and 15% from 2010 through 2011 (*P* value for trend, < .001) (Table 2). Even restricting analyses to those products that were reformulated, the magnitude of TFA reduction also declined over time (*P* for trend < .001). Among products that reduced TFA during each period, the average reduction was 75% (1.5 [SD, 1.3] g/serving decline) during 2007 and 2008, compared with 61% (1.0 [SD, 0.7] g/serving decline) during 2010 and 2011 (*P* = .009 comparing 2007–2008 with 2010–2011). The average TFA reduction from 2008 through 2010 was intermediate. Similarly, restricting to products containing 0.5 or more g per serving TFA at the start of each period, the pace of percentage decline in TFA slowed across years (*P* value for trend = .02) (Table 2).

Characteristic	Year			
	2007	2008	2010	2011
Number of products containing any TFA (%)	270 (100)	231 (86)	203 (75)	197 (72)
Mean (SE) TFA content, g/serving	1.9 (0.1)	1.3 (0.1)	1.0 (0.1)	0.9 (0.1)
Median (IQR) TFA content, g/serving	1.5 (1.0–2.5)	1.0 (0.25–2.0)	0.3 (0.3–1.5)	0.3 (0-1.5)

Among the 31 products that reduced TFA from 2010 through 2011 (average reduction 1.0 [SD, 0.7] g/serving), the average SFA content was also reduced by 0.4 (SD, 1.5) g per serving, for an overall decrease in combined TFA and SFA of 1.4 (SD, 1.6) g per serving. Among the 15 products that increased TFA during 2010 and 2011 (average increase, 0.7 [SD, 0.5] g/serving), the average SFA content also increased by 0.2 (SD, 2.2) g per serving, and the total combined TFA and SFA content by 0.9 (SD, 2.1) g per serving.

**Figure 2:**
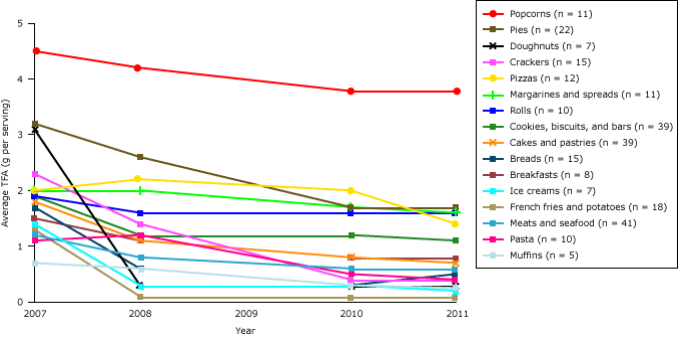
Average trans fatty acid (TFA) content from 2007 through 2011 of brand-name US supermarket food products that contained ≥0.5 g/serving TFA in 2007, by food categories. Data were not collected in 2009. All products listing 0 g TFA but still containing partially hydrogenated oils in the ingredients list were considered to still contain 0.25 g per serving of TFA. Abbreviation: SD, standard deviation. **Table:** 

Among food categories, the largest gram per serving declines in TFA were evident for doughnuts (−2.8 g/serving), crackers (−1.9 g/serving), and pies (−1.4 g/serving), and the largest percentage declines were seen in French fries/other potatoes (88% reduction), doughnuts (81%), and ice creams (73%), from 2007 through 2011 (Figure 2) (see supplementary Table 2, Appendix B). The smallest percentage declines were seen for rolls (15%), margarines (19%), and popcorns (19%). In all years, popcorn products contained the most TFA; popcorn products contained an average of 4.5 g per serving in 2007 and 3.8 g per serving in 2011, representing a modest decline (0.7 g/serving). In comparison, all 18 French fries/other potato products containing TFA in 2007 were reformulated to 0.5 g or less TFA per serving by 2008 and remained at this level through 2011. By 2011, only 5 of these 18 French fries/other potatoes contained PHVO and IP-TFA, while 13 had completely eliminated those substances. All 7 doughnut products and all 5 muffin products were also reformulated by 2011 to contain less than 0.5 g per serving TFA, with large gram per serving declines for doughnuts (−2.8 g/serving) and smaller declines for muffins (−0.3 g/serving). All 12 of these doughnut and muffin products continued to contain PHVO as an ingredient in 2011, indicating they still contained small amounts of TFA.

Food Product	TFA Content in g/Serving by Year, Mean (SD)			
	2007	2008	2010	2011
Breads (n = 15)	1.7 (0.7)	0.6 (0.8)	0.3 (0.6)	0.5 (0.8)
Breakfasts (n = 8)	1.5 (0.9)	1.1 (0.9)	0.8 (1.0)	0.8 (1.0)
Cakes and pastries (n = 39)	1.8 (1.3)	1.1 (1.0)	0.8 (0.9)	0.7 (0.7)
Cookies, biscuits, and bars (n = 39)	1.9 (1.0)	1.2 (1.1)	1.2 (1.2)	1.1 (1.1)
Crackers (n = 15)	2.3 (1.3)	1.4 (1.3)	0.4 (0.5)	0.4 (0.5)
Doughnuts (n = 7)	3.1 (1.8)	0.3 (0)	0.3 (0)	0.3 (0)
French fries and potatoes (n = 18)	1.3 (0.6)	0.1 (0.3)	0.1 (0.1)	0.1 (0.1)
Ice creams (n = 7)	1.4 (1.3)	0.3 (0.3)	0.3 (0.3)	0.2 (0.2)
Margarines (n = 11)	2.0 (0.6)	2.0 (0.5)	1.7 (0.9)	1.6 (0.8)
Meats and seafood (n = 41)	1.2 (0.9)	0.8 (0.6)	0.6 (0.6)	0.6 (0.6)
Muffins (n = 5)	0.7 (0.3)	0.6 (0.5)	0.3 (0.1)	0.25 (0)
Pasta (n = 10)	1.1 (0.8)	1.2 (1.2)	0.5 (0.6)	0.4 (0.4)
Pies (n = 22)	3.2 (1.1)	2.6 (1.4)	1.7 (1.6)	1.7 (1.6)
Pizzas (n = 12)	2.0 (1.6)	2.2 (1.5)	2.0 (1.6)	1.4 (1.5)
Popcorns (n = 11)	4.5 (1.3)	4.2 (1.8)	3.8 (2.1)	3.8 (2.2)
Rolls (n = 10)	1.9 (0.6)	1.6 (1.0)	1.6 (0.8)	1.6 (0.8)

**Figure 3:**
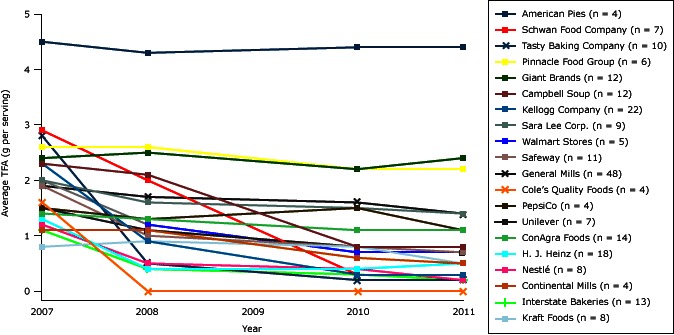
Average trans fatty acid (TFA) content from 2007 through 2011 of 236 brand-name US supermarket food products that contained ≥0.5 g/serving TFA in 2007, by their major (>3 products) parent companies. Data were not collected in 2009. All products listing 0 g TFA but still containing partially hydrogenated oils in the ingredients list were considered to still contain 0.25 g per serving of TFA. Abbreviation: IQR, interquartile range. **Table:** 

Among all 270 products, changes in TFA content were different by company or manufacturer (Figure 3) (see supplementary Table 3, Appendix B). These products represented 48 parent companies, with the most from General Mills (48 products), Kellogg (22 products), and H. J. Heinz (18 products) (Appendix A). Among the 20 companies having at least 4 TFA-containing products in 2007, the largest percentage declines in TFA occurred in products from Cole’s Quality Foods (100%), Schwan Food Company (90%), and Tasty Baking Company (87%), and the smallest declines were in foods made by American Pies (3%), Giant Foods (12%), and ConAgra Foods (13%). The largest gram per serving declines occurred in products from Schwan Food Company (2.6 g/serving) and Tasty Baking Company (2.5 g/serving), down to average TFA contents of 0.3 g and 0.2 g per serving, respectively. In all years, products from American Pies contained the most average TFA (>4 g/serving), with little decline between 2007 and 2011. In all years, products from Pinnacle Foods and Giant contained on average more than 2.0 g per serving TFA, with little change by 2011.

Supermarket Product Parent Company (no. of Products)	TFA Content in g/serving by Year			
	2007	2008	2010	2011
American Pies (n = 4)	4.5 (0.4)	4.3 (0.6)	4.4 (0.3)	4.4 (0.3)
Campbell Soup (n = 12)	2.3 (1.6)	2.1 (1.7)	0.8 (1.1)	0.8 (0.9)
Cole’s Quality Foods (n = 4)	1.6 (0.5)	0 (0)	0 (0)	0 (0)
ConAgra Foods (n = 14)	1.4 (0.5)	1.3 (0.6)	1.1 (0.8)	1.1 (0.7)
Continental Mills (n = 4)	1.1 (0.6)	1.1 (0.6)	0.6 (0.9)	0.5 (0.7)
General Mills (n = 48)	1.9 (1.0)	1.7 (1.2)	1.6 (1.2)	1.4 (1.1)
Giant Brands (n = 12)	2.4 (1.8)	2.5 (1.8)	2.2 (2.0)	2.4 (2.0)
H. J. Heinz (n = 18)	1.3 (0.6)	0.4 (0.5)	0.4 (0.5)	0.5 (0.6)
Interstate Bakeries (n = 13)	1.1 (0.6)	0.4 (0.4)	0.3 (0.2)	0.2 (0.1)
Kellogg Company (n = 22)	2.3 (1.4)	0.9 (1.3)	0.3 (0.5)	0.3 (0.5)
Kraft Foods (n = 8)	0.8 (0.3)	0.9 (0.4)	0.8 (0.3)	0.5 (0.4)
Nestlé (n = 8)	1.2 (1.2)	0.5 (0.6)	0.4 (0.7)	0.2 (0.2)
PepsiCo (n = 4)	1.5 (0.7)	1.3 (0.3)	1.5 (0.7)	1.1 (0.3)
Pinnacle Food Group (n = 6)	2.6 (1.6)	2.6 (1.6)	2.2 (1.7)	2.2 (1.7)
Safeway (n = 11)	1.9 (1.2)	1.0 (1.2)	0.8 (1.1)	0.7 (1.2)
Sara Lee Corp. (n = 9)	2.0 (0.9)	1.6 (1.3)	1.5 (1.3)	1.4 (1.3)
Schwan Food Company (n = 7)	2.9 (1.2)	2.0 (1.5)	0.3 (0.4)	0.3 (0.4)
Tasty Baking Company (n = 10)	2.8 (2.2)	0.5 (0.7)	0.2 (0.1)	0.2 (0.1)
Unilever (n = 7)	1.5 (1.0)	1.1 (1.0)	0.8 (0.9)	0.7 (0.8)
Walmart Stores (n = 5)	2.0 (0.4)	1.2 (1.2)	0.7 (1.3)	0.7 (1.3)

This variation in reformulations across companies was not simply due to differences in the types of products they sold across food categories. The observed differences persisted in analyses stratified by food category and parent company. For example, among identified popcorn products, the TFA content of Safeway’s product fell from 4.5 to 0 g per serving from 2007 through 2011, while the General Mills product increased from 5 g to 6 g per serving during the same period. (see supplementary Figures 1–16, Appendix B for complete stratified analysis by food categories and by parent companies). By 2011, only 3 of the 20 major companies (Cole’s Quality Foods, Interstate Bakeries, Tasty Baking Company) had reformulated all of their products identified in this analysis to contain less than 0.5 g per serving TFA. Of these, only Cole’s Quality Foods had completely eliminated PHVO from its 4 bread-based products in 2011. Among the 28 other companies having fewer than 4 TFA-containing products in our 2007 survey, 16 companies had reformulated all their products to contain less than 0.5 g per serving TFA, and 6 of these companies had fully eliminated PHVO in all their products by 2011.

In sensitivity analyses, we further evaluated the other 90 TFA-containing products that were discontinued by 2011. Across major food categories, the proportion of discontinued products varied from 0% to 56% (see supplementary Table 4, Appendix B). To test the theoretically largest trends in reformulations over time, we assumed that these discontinued products were “reformulated” to contain no PHVO and 0 g TFA (see supplementary Tables 5–6, Appendix B). As expected, estimated magnitudes of TFA declines were modestly larger. For instance, the theoretical average TFA content of all 360 foods was calculated to be 0.7 (SD, 1.1) g per serving in 2011, compared with the actual 0.9 (SD, 1.2) g per serving of the 270 foods still on the market. However, our findings for the numbers and amounts of TFA reformulations, and the slowing of reformulations over time, were similar.

## Discussion

In this analysis of major US store brand and brand-name food products containing TFA in 2007, we found that two-thirds (66%) were reformulated to reduce TFA by 2011. Among reformulated products, 82% reduced TFA to less than 0.5 g per serving, although only half of these had completely eliminated IP-TFA. Of all products, reformulation eliminated an average of approximately 1 g of TFA per serving, representing an average percentage reduction of 49%. These findings suggest that some food manufacturers, supported by oil processors, seed developers, and farmers, have made substantial progress in recent years in replacing PHVO with alternative oils.

Yet, both the pace of reformulation and magnitude of TFA reduction significantly decreased over time. This slowing reformulation was related to both little reformulation in certain food categories and failure of certain parent companies or manufacturers to embrace reformulation to replace PHVO with more healthful oils. For example, we identified substantial heterogeneity in TFA reformulations by both type of food product and parent company.

Differences in reformulation could partly reflect technical challenges of reformulating certain foods. Most French fries or other potato products, ice creams, and doughnuts were reformulated to contain less than 0.5 g per serving TFA by 2011, while foods in other food categories, such as popcorns, pies, margarines, and rolls, still averaged more than 1.5 g per serving TFA. However, in each of the latter food categories, we also identified individual products that reduced TFA to less than 0.5 g per serving. These findings suggest it is possible to produce marketable forms of these products with reduced or eliminated TFA; therefore, reasons other than technical difficulties with reformulation may be contributing to remaining high TFA contents in these types of products.

Differences in TFA reformulations among companies were also identified. Some manufacturers markedly reduced TFA contents of their products while others showed little evidence of progress. Our investigation indicated instances in which TFA reformulation efforts have been successful, both in food categories and among parent companies, and identified other food categories and companies where additional focus on reformulation strategies would be most relevant. For example, a recent study using data from the 1999–2002 NHANES survey listed French fries, popcorns, pies, and margarines among the top 10 food group contributors to TFA intake in the US population during that period (12). Our more contemporary analysis suggests that most French fry manufacturers have subsequently made strides toward eliminating IP-TFA, while popcorns and pies still require intensive reformulation efforts. Our results also indicate that, although TFA consumption in the United States decreased from 2006 through 2009 (8,9), many major national products in the food supply continue to contain high amounts of TFA, and reformulations have slowed after 2008 and especially after 2010.

By 2011, 73 (50%) of the 146 products listing 0 g TFA on the Nutrition Facts panel still listed PHVO in the ingredients panel. This indicates continued presence of IP-TFA in these 73 products, theoretically up to 0.49 g per serving. However, the average reduction in TFA in these products was still large: 1.4 g per serving. This finding suggests that consumers can view a “0 g TFA” listing on a reformulated product with cautious optimism, as a substantial proportion of TFA content has been eliminated. Nonetheless, when consumed across multiple servings and different products, these remaining amounts of PHVO pose a health risk and should be eliminated. Given the many products that were not reformulated at all, our findings suggest that public health attention should focus on reformulation of current high-TFA foods; additional methods to fully eliminate TFA in foods with less than 0.5 g TFA can follow.

Our study has limitations. The observed trends in TFA reformulations are specific to the 360 identified products, and these foods did not represent an exhaustive accounting of every possible TFA-containing food in the United States or a random sampling of all products made by all companies. Certain food categories, such as frosting, may have been underrepresented in our initial survey. However, our large sample from major US grocers likely provides a reasonable national representation of major packaged food products in the United States and of progress in TFA reformulation in these foods from 2007 through 2011. In addition, our investigation represents the most comprehensive contemporary assessment of TFA reformulations of packaged foods sold in the United States. We evaluated total TFA above 0.5 g per serving, as current labeling methods do not distinguish between industrial and natural sources. Some TFA on the Nutrition Facts panel could represent TFA from ruminant-derived products or nonhydrogenated vegetable oils (eg, due to deodorization). However, ruminant TFA would represent a small proportion of total TFA at or above 0.5 g per serving; below 0.5 g per serving, we based our assessment on the presence of PHVO. We did not assess new products introduced after 2007, some of which could contain TFA. However, a recent US Department of Agriculture evaluation of new products introduced between 2006 and 2010 found a minority of new products (5.3%) containing TFA, with decreasing introductions over time (13). Inclusion of these new products could increase the absolute exposure of the US population to TFA but would not alter our conclusions regarding reformulations of existing TFA-containing products. Nonetheless, even these new product introductions will further slow the pace of elimination of IP-TFA from the food supply, and introduction of new products that contain IP-TFA should be eliminated. We also did not collect any information on the prices of the products, which may have changed over time.

Our findings suggest that some food manufacturers have made progress in reducing TFA in their US products, but substantial variation exists by food type and by parent company, and overall progress has significantly slowed over time. Our findings also indicate that, even among this subset of national products, many products remain in the food supply that contain substantial amounts of IP-TFA. Because TFA consumption is harmful even at low levels (1,14), our results emphasize the need for continued major efforts and commitment toward reformulating (or discontinuing) foods to eliminate PHVO, with particular focus on certain food categories and companies. The heterogeneity of success also suggests that nationally coordinated policy efforts, including both voluntary and regulatory strategies and surveillance of key foods and companies, would be most effective to eliminate exposure to IP-TFA in the general population. For instance, the Food and Drug Administration could solve the problem expeditiously by declaring PHVO as no longer “generally recognized as safe,” thereby placing limits on the presence of IP-TFA in foods (small amounts occur when vegetable oil is purified), as several other nations including Austria, Denmark, Iceland, Sweden, and Switzerland have done (15).

## Appendix A. Major US Brand-Name Food Products (N = 270) that Contained ≥0.5 g/serving TFA in 2007 and Were Still Marketed in 2011

**Table:**

Food Product	US Brand Name
Breads (n = 15)	Cole Big Texan Texas Toast; Cole Cheesesticks; Cole Garlic Bread Mini Loaf; Cole Garlic Breadsticks; Eggo Cinnamon Toast; Goya Tamales; Old El Paso Fajita Dinner Kit (dry, boxed); Old El Paso Gordita Dinner Kit (dry, boxed); Old El Paso Stand N Stuff Yellow Corn Taco Shells; Pepperidge Farm 5 Cheese Garlic Bread; Pepperidge Farm Garlic Bread; Pepperidge Farm Texas Toast; Pillsbury Breadsticks; Sara Lee Croissants (French Style); T.G.I. Friday Mozzarella Snack Sticks
Breakfasts (n = 8)	Aunt Jemima Croissant Sandwiches (sausage, egg, and cheese variety); Bob Evans Snackwiches (sausage, egg, and cheese biscuit); Jimmy Dean Breakfast Bowls (bacon variety); Jimmy Dean Breakfast Skillets; Jimmy Dean Croissant Sandwiches (ham and cheese variety); Nancy’s Quiche Lorraine; Pillsbury Toaster Scrambles (cheese, egg, and bacon variety); Red Baron Biscuit Style Scrambles with Bacon
Cakes and pastries (n = 39)	Betty Crocker Cinnabon Cinnamon Streusel Mix; Betty Crocker Complete Desserts Triple Chocolate Hot Fudge Cake; Betty Crocker Gingerbread Mix (blueberry variety); Betty Crocker Pound Cake Mix; Betty Crocker Quick Bread (banana and cinnamon streusel variety); Betty Crocker Super Moist Cake Mix; Betty Crocker Warm Delights; Betty Crocker Whipped Frosting; Drake’s Devil Dogs Snack Cakes; Drake’s Ring Dings Snack Cakes; Drake’s Yodels Snack Cakes; Duncan Hines Creamy Home Frosting; Ghirardelli Brownie Mix; Great Value No Bake Cheesecake Mix; Hostess Cupcakes; Hostess HoHo Snack Cakes; Hostess Honey Bun with icing; Hostess Suzy Q’s Snack Cakes; Hostess Zingers Snack Cakes (vanilla and devil’s food varieties); Hostess Zingers Snack Cakes (raspberry variety); Krusteaz Cinnamon Crumb Cake Mix; Little Debbie Swiss Cake Rolls; Pepperidge Farm Chocolate Fudge 3-Layer Cake; Pepperidge Farm Coconut 3-Layer Cake; Pepperidge Farm Fudge Stripe Cake; Pepperidge Farm Golden 3-Layer Cake; Pepperidge Farm Puff Pastry Sheets; Pepperidge Farm Puff Pastry Shells; Pillsbury Creamy Supreme Reduced Sugar Frosting (Vanilla); Pillsbury Toaster Strudel; Sara Lee Original Cream Cheesecake (strawberry and classic varieties); Sara Lee Pound Cake; Tastykake Butterscotch Krimpets; Tastykake Chocolate Cupcakes; Tastykake Crème Filled Chocolate Cupcakes; Tastykake Crème Filled Koffee Cake Cupcakes; Tastykake Crème Filled Vanilla Cupcakes; Tastykake Glazed Honey Bun; Tastykake Sensables Sugar Free Kreme Filled Chocolate Cake
Cookies, biscuits, and bars (n = 39)	Archway Frosty Lemon Cookies; Archway Iced Oatmeal Cookies; Betty Crocker Sugar Cookie Mix; Betty Crocker Sunkist Lemon Bar Mix; Famous Amos Chocolate Chip Cookies; Famous Amos Chocolate Chip Pecan Cookies; Famous Amos Vanilla Sandwich Cookies; Frito Lay Grandma’s Peanut Butter Sandwich Crème Cookies; Frito Lay Grandma’s Vanilla Sandwich Crème Cookies; Giant Jumbos Buttermilk Biscuits; Giant Jumbo Flakey Biscuits; Goetze’s Caramel Cream Candies; Jimmy Dean Biscuit Sandwiches; Keebler Animal Cookies; Keebler Chips Deluxe Cookies; Keebler E.L. Fudge Cookies; Keebler Fudge Shoppe Fudge Sticks; Keebler Fudge Shoppe Fudge Stripe Cookies; Keebler Gripz Chips Deluxe Cookies; Keebler Soft Batch Cookies; Keebler Vienna Finger Cookies; Krusteaz Key Lime Bar Mix; Pillsbury Big Deluxe Cookie Dough (Chocolate Chip); Pillsbury Big Deluxe Cookie Dough (Flag/Special Edition); Pillsbury Create & Bake Cookie Dough (chocolate chip and sugar variety); Pillsbury Create & Bake Cookie Dough (peanut butter variety); Pillsbury Golden Layer Buttermilk Biscuits; Pillsbury Grands! Biscuits 12 Southern Style–Freezer to Oven; Pillsbury Grands! Flakey Layer Biscuits; Pillsbury Grands! Homestyle Biscuits; Pillsbury Ready to Bake Cookie Dough (chocolate chip); Pillsbury Ready to Bake Cookie Dough (sugar cookie); Safeway Chocolate Chewy Granola Bar; Safeway Chocolate Pudding Snacks; Safeway Jumbos Butter Flavor Biscuits; Safeway Fudge Stick Cookies; Safeway Vanilla Crème Wafer Cookies; Safeway Swirl Cookies; Tollhouse Sugar Cookie Dough
Crackers (n = 15)	Austin Grilled Cheese Crackers; Austin Cheese Crackers with Peanut Butter; Austin Cheese Crackers with Cheddar Cheese; Austin Peanut Butter and Jelly Cracker; Frito Lay Peanut Butter on Toast Crackers; Frito Lay Peanut Butter on Cheese Crackers; Gardetto’s Snack Mix; Keebler Cheese and Peanut Butter Crackers; Keebler Club and Cheddar Sandwich Crackers; Keebler Graham Cracker Crumbs; Keebler Toast and Peanut Butter Crackers; Keebler Town House Bistro Multigrain Crackers; Safeway Chocolate Crackers; Safeway Woven Wheat Crackers; Stouffers Whales Snack Crackers
Doughnuts (n = 7)	Hostess Donettes Donuts; Hostess’ Donuts (assorted with frosting); Krispy Kreme Krispy Juniors Donuts; Krispy Kreme Mini Crullers Doughnuts; Krispy Kreme Original Glazed Doughnuts (yeast variety); Tastykake Powdered Sugar Mini Donuts (cake variety); Tastykake Rich Frosted Mini Donuts (cake variety)
French fries/other potatoes (n = 18)	Betty Crocker Au Gratin Potatoes; Betty Crocker Scalloped Potatoes; Giant Potato Skins (cheddar, bacon, and nacho variety); Great Value Onion Rings; Great Value Regular Cut French Fries; Idahoan Baby Reds Mashed Potatoes; Idahoan Four Cheese Mashed Potatoes; Mrs. T’s Pierogies (potato, broccoli, and cheddar variety); Ore Ida Easy Fries; Ore Ida Extra Crispy Golden Crinkles Fries; Ore Ida Fast Food Fries; Ore Ida Golden Fries; Ore Ida Seasoned Crinkles Fries; Ore Ida Zesties Fries; Simply Potatoes Garlic Mashed Potatoes; Simply Potatoes Mashed Potatoes; Simply Potatoes Mashed Sweet Potatoes; T.G.I. Friday’s Potato Skins
Ice creams (n = 7)	Drumstick Simply Dipped Vanilla Ice Cream Cone; Good Humor Chocolate Éclair Ice Cream bar; Good Humor Giant King Cone Ice Cream Cone; Good Humor Giant Vanilla Ice Cream Sandwich; Good Humor Toasted Almond Ice Cream Bars; Simply Enjoy Chocolate Coated Crème Puffs; Tollhouse Chocolate Chip Cookie and Vanilla Ice Cream Sandwich
Margarines and spreads (n = 11)	Blue Bonnet Regular Stick Margarine; Canoleo Tub Margarine; Fleischmann’s Stick Margarine; Giant Soft Spread Tub Margarine; Giant Stick Margarine; Great Value Stick Margarine; I Can’t Believe It’s Not Butter Stick Margarine; Imperial Stick Margarine; Land O’Lakes Stick Margarine; Parkay Original Stick Margarine; Shedd’s Spread Country Crock Stick Margarine
Meats and seafood (n = 41)	Banquet Chicken Wings (hot and spicy variety); Banquet Crispy Chicken Variety Pack; Banquet Meat Loaf meal; Barber Foods Stuffed Chicken Breast Kiev; Boston Market Roasted Pork (frozen dinner); Boston Market Salisbury Steak (frozen dinner); Boston Market Turkey Breast Medallions; Boston Market Turkey Breast Medallions with stuffing; Campbell’s Chunky Chili (spicy beef and bean variety); ChiChi’s Taco Sauce with Seasoned Ground Beef; Contessa Shrimp Primavera; Contessa Shrimp Scampi; Contessa Shrimp Stir Fry; Delimex Chicken Quesadillas; Don Miguel Beef and Cheese Empanadas; Hamburger Helper Complete Meals (cheesy chicken) (dry boxed); Hormel Beef Tips with Gravy; Kid Cuisine All American Fried Chicken Frozen Dinner; Mama Lucia Homestyle Meatballs (boxed, frozen); Margaritaville Island Lime Shrimp; Margaritaville Jammin’ Jerk Shrimp (frozen); Marie Callender’s Country Fried Beef Steak Dinner; Marie Callender’s Pot Pie (beef variety); Marie Callender’s Pot Pie (chicken variety); Neptune Shrimp Scampi; On-Cor Parmagiana; On-Cor Gravy and Six Salisbury Steaks Frozen Dinner; On-Cor Stuffed Cabbage Rolls with Beef; Oscar Mayer Lunchables Deluxe Turkey and Ham with Swiss and Cheddar; Oscar Mayer Lunchables Turkey and American Stacker; Slim Jim Monster Stick Original Meat Stick; Slim Jim Pepperoni Meat Stick; Stouffer’s Chicken Enchiladas with Cheese Sauce and Rice; Stouffer’s Fish Fillet Meal; Stouffer’s Pot Pie (white meat chicken variety); Stouffer’s Skillet Yankee Pot Roast (contains potatoes); T.G.I. Friday’s Buffalo Wings; T.G.I. Friday’s Mexican style Chicken Quesadillas; T.G.I. Friday’s Steak Quesadilla Rolls; T.G.I. Friday’s Sweet and Smokey Popcorn Chicken; White Castle Hamburgers
Muffins (n = 5)	Betty Crocker Muffin Mix; Betty Crocker Sunkist Lemon Poppy Seed Bread and Muffin Mix; Duncan Hines Bakery Style Muffin Mix (blueberry streusel variety); Krusteaz Wild Blueberry Muffin Mix; Sun-Maid Honey Raisin Bran Muffin Mix
Pasta (n = 10)	Campbell’s Spaghetti O’s; Great Value Premium Macaroni and Cheese (dry box); Hamburger Helper Chili Cheese Meal; Hamburger Helper Microwave Single Meals (cheeseburger macaroni); La Choy Chow Mein Noodles (dry, bagged salad topping); Michelina’s Cheese Ravioli; Michelina’s Macaroni and Cheese with Ham; Safeway Deluxe Macaroni and Cheese Dinner; Safeway/Vons Shells and Cheese Dinner (dry box); Stouffer’s Lasagna with Meat and Sauce
Pies (n = 22)	Betty Crocker Pie Crust Mix; Edwards Banana Crème Pie; Edwards Georgia Pecan Pie; Edwards Key Lime Pie; Edwards Oreo Crème Pie; Edwards Turtle Pie; Giant Graham Cracker Pie Crust; Hostess Fruit Pies; Keebler Ready Crust (chocolate and graham varieties); Keebler Ready Crust–Reduced Fat Graham Crust; Little Debbie Apple Pie; Marie Callender’s Cherry Crunch Pie; Marie Callender’s Chocolate Satin Pie; Marie Callender’s Deep Dish Pie Shells; Marie Callender’s Dutch Apple Pie; Marie Callender’s Razzleberry Pie; Mrs. Smith’s Lemon Meringue Pie; Pepperidge Farm Apple Turnovers; Pillsbury Turnovers (apple variety); Sara Lee Chocolate Mint Crème Pie; Sara Lee Key West Lime Pie; Tastykake Fruit Pies
Pizzas (n = 12)	California Pizza Kitchen Crispy Thin Crust BBQ Chicken Pizza; California Pizza Kitchen Crispy Thin Crust Margarita Pizza; California Pizza Kitchen Crispy Thin Crust Sicilian Pizza; California Pizza Kitchen Crispy Thin Crust White Pizza; Celeste Pizza for 1 (4-cheese variety); Celeste Pizza for 1 (original variety); Celeste Pizza for 1 (Pepperoni variety); DiGiorno Garlic Bread Pizza (4-cheese variety); DiGiorno Thin Crispy Crust Pizza (4-cheese variety); Totino’s Crispy Crust Party Pizza (cheese variety); Totino’s Crispy Crust Party Pizza (sausage and pepperoni variety); Totino’s Pepperoni Pizza Rolls
Popcorns (n = 11)	Giant Kapop Kettle Korn Popcorn; Giant Kapop Natural Light Popcorn; Giant Kapop Ultimate Butter Popcorn; Jolly Time Blast O Butter Popcorn; Jolly Time Sassy Salsa Popcorn; Pop Secret 1 Step Cheddar Popcorn; Pop Secret Homestyle Popcorn; Pop Secret Old Fashioned Kettle Corn Popcorn; Pop Weaver Butter Popcorn; Safeway Giant Pop Extra Butter Microwave Popcorn; Utz Hulless Puff’n Corn
Rolls (n = 10)	Giant Crescent Dinner Rolls; Giant Reduced Fat Crescent Rolls; Hostess Cinnamon Sweet Rolls; Pillsbury Cinnamon Rolls with Icing; Pillsbury Crescent Rolls (big and flaky variety); Pillsbury Crescent Rolls (original and garlic butter variety); Pillsbury Grands! Cinnamon Rolls with icing; Pillsbury Orange Sweet Rolls; Pillsbury Sweet Rolls Cinnamon Mini Bites; Pillsbury Traditional Dinner Rolls

## Appendix B. Stratified Analysis of TFA Content, by Food Categories and Parent Companies

Supplemental figures:

Supplemental tables:
