# Supplementary material for: Trends in Trans Fatty Acids Reformulations of US Supermarket and Brand-Name Foods From 2007 Through 2011
Source: Prev Chronic Dis. 2013 May 23;10:E85. doi: 10.5888/pcd10.120198 (PMC3670643; doi:10.5888/pcd10.120198)

Breads


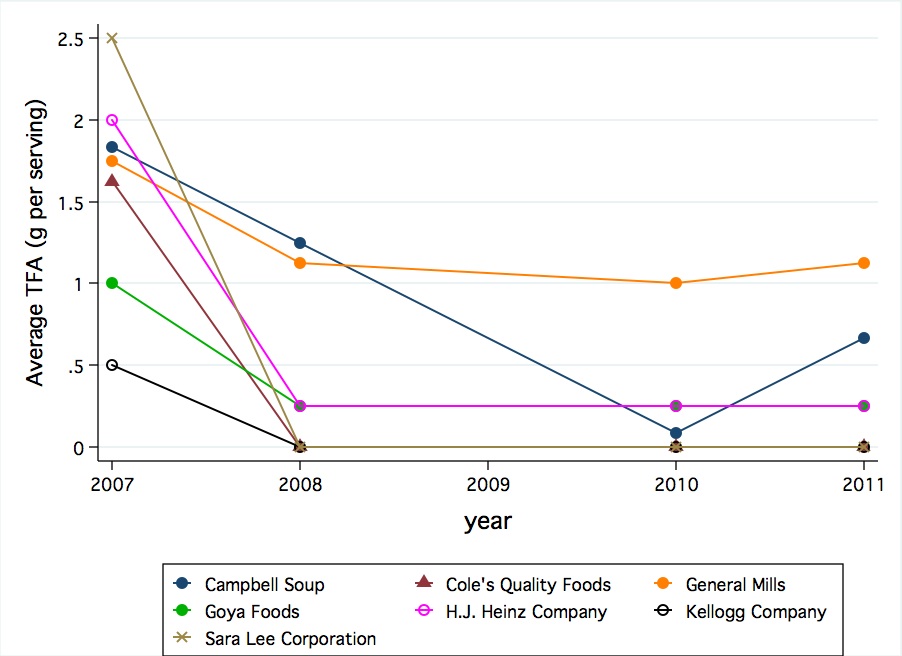


Breakfasts


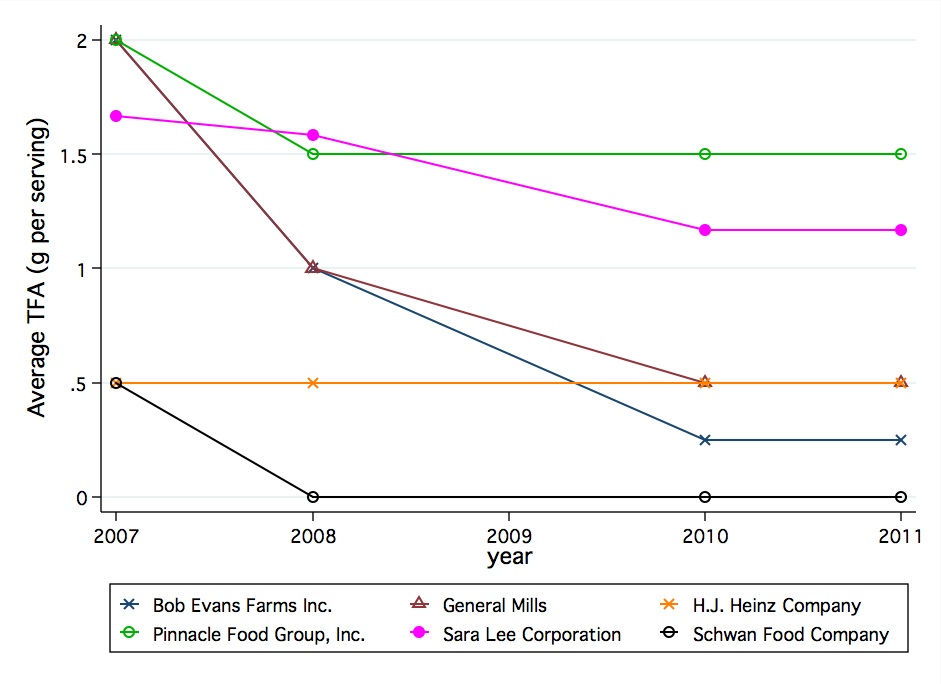


Cakes and Pastries


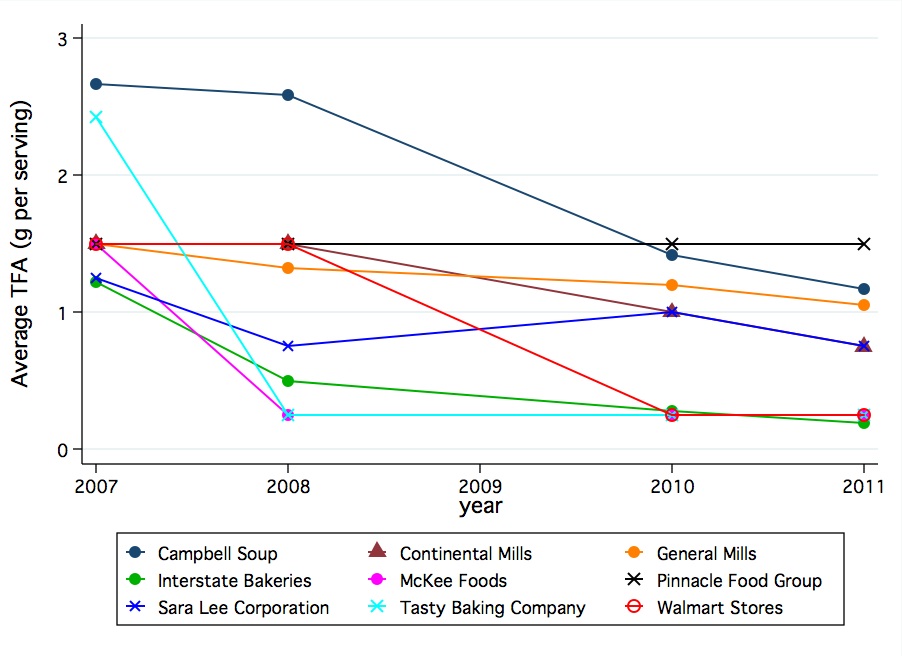


Cookies, Biscuits, and Bars


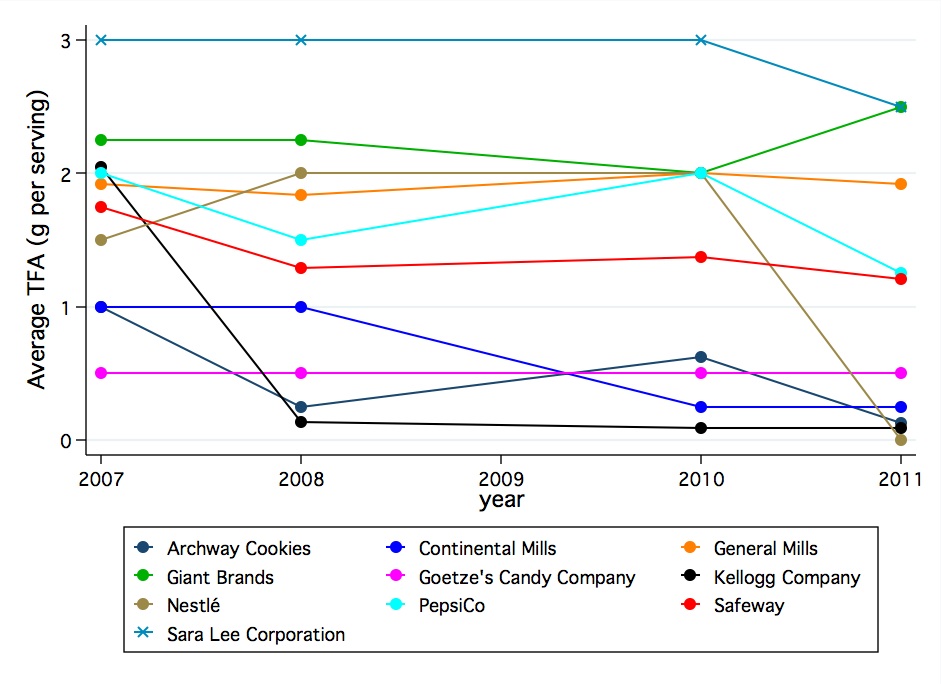


Crackers


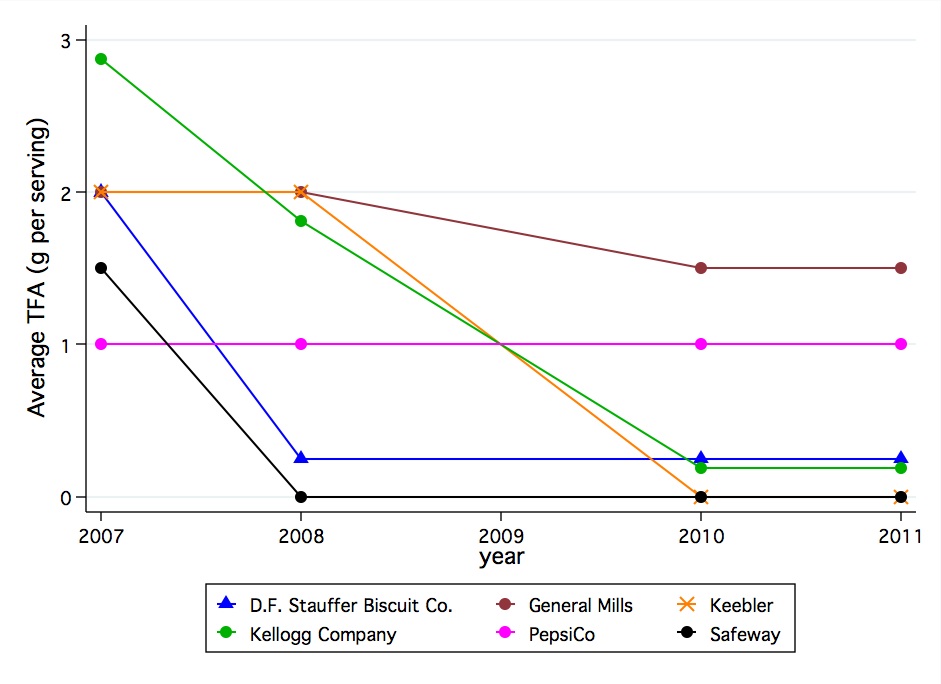


Doughnuts


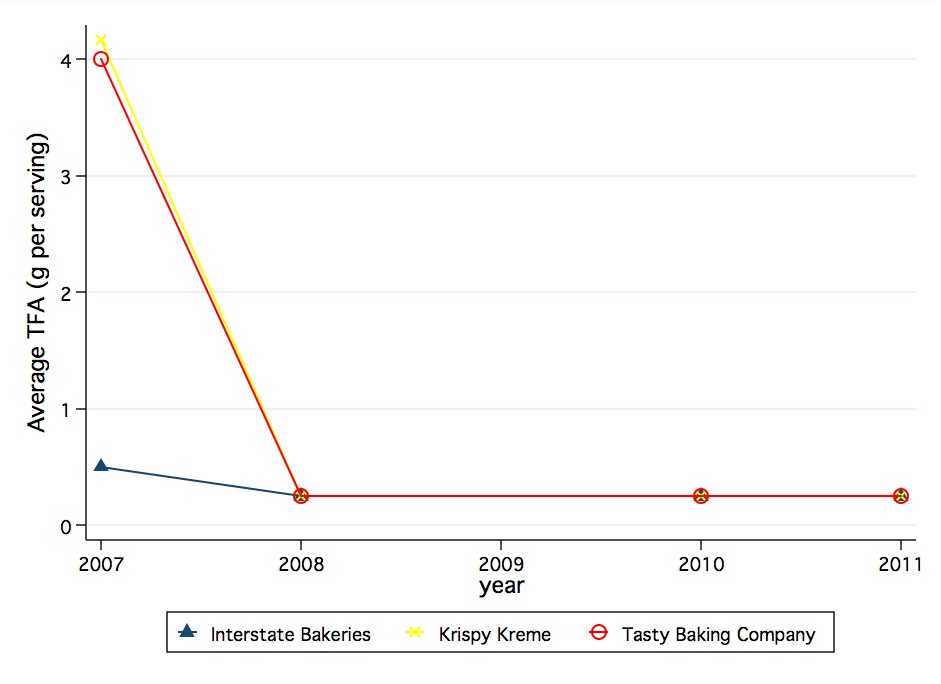


French Fries and Potatoes


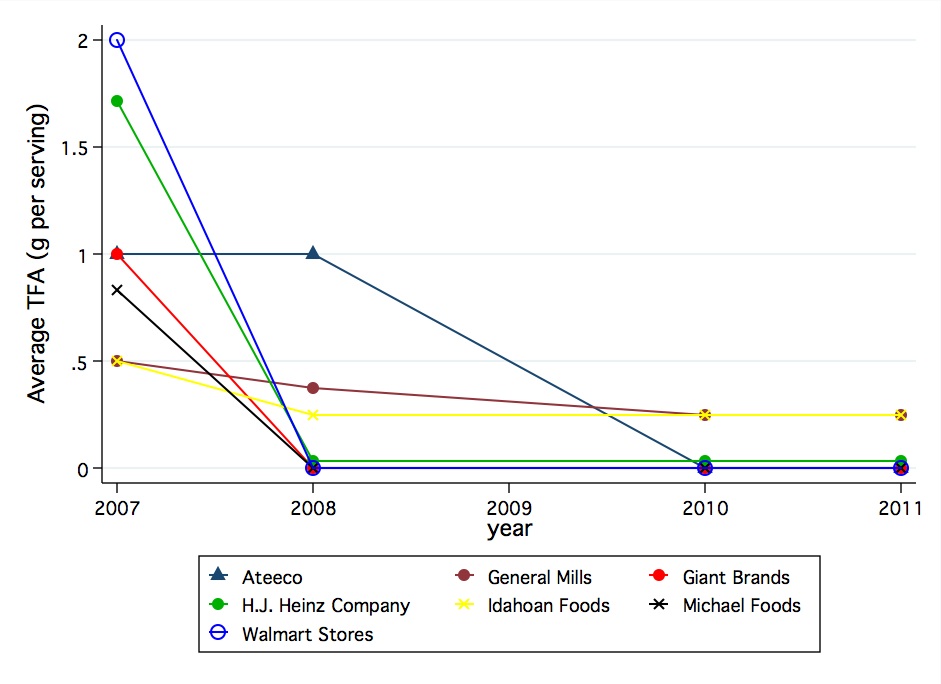


Ice Creams


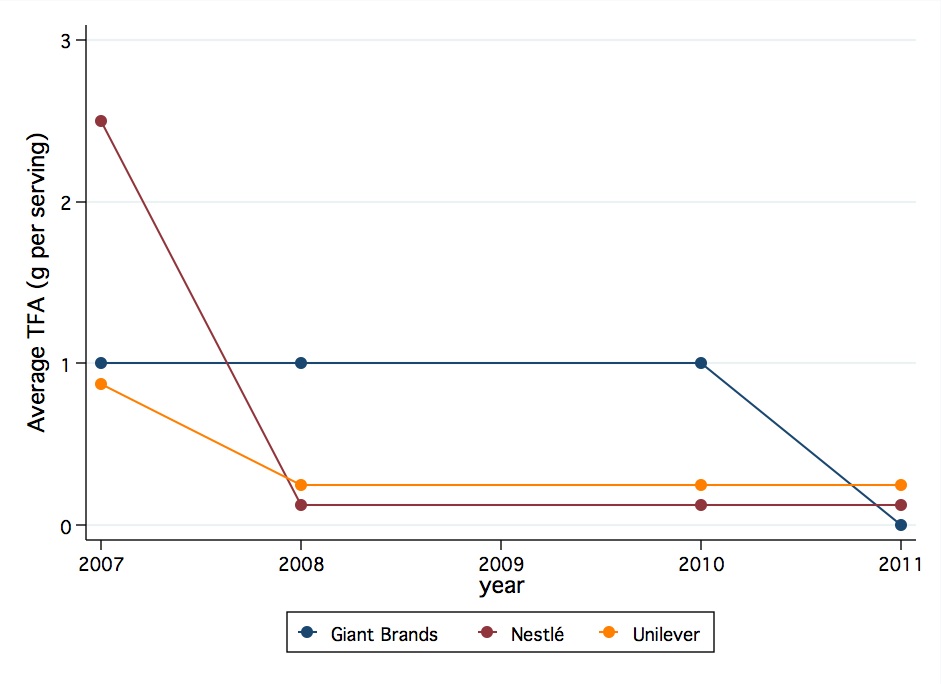


Margarines


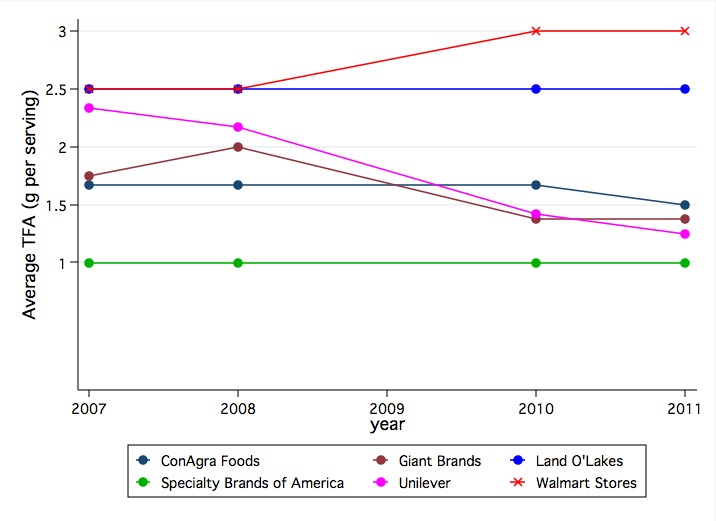


Meats and Seafood


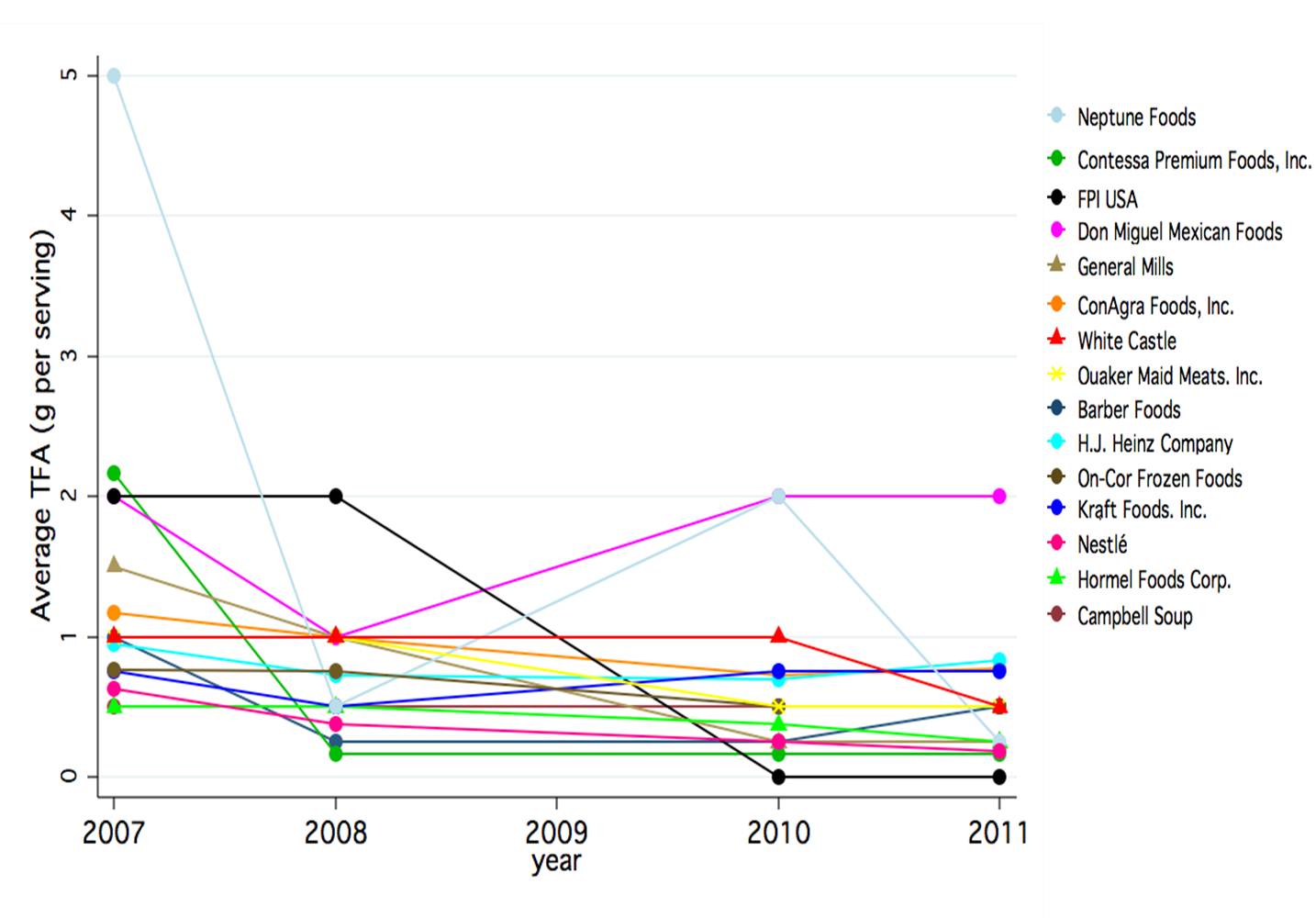


Muffins


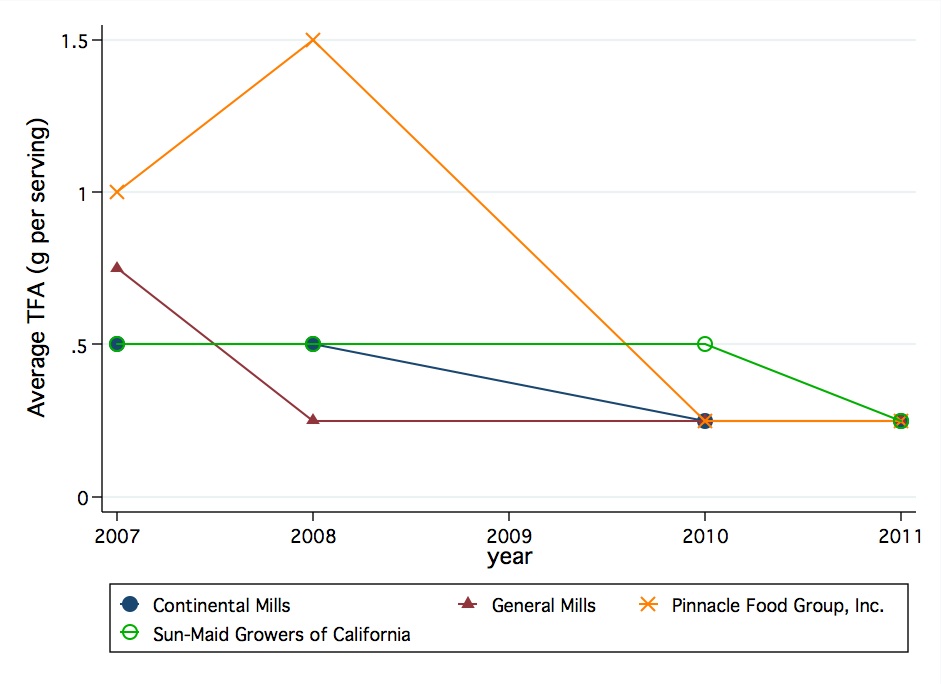


Pasta


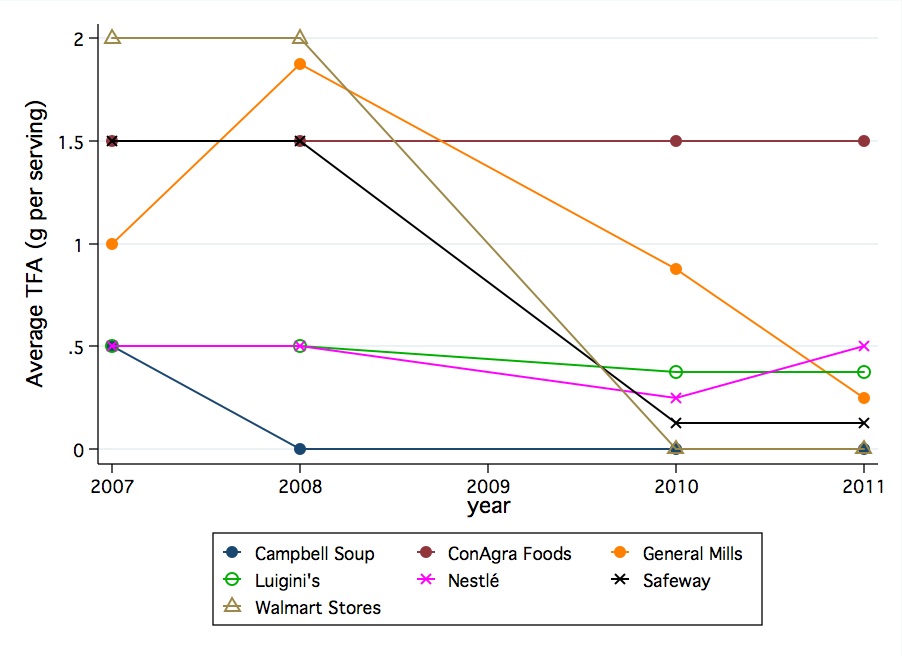


Pies


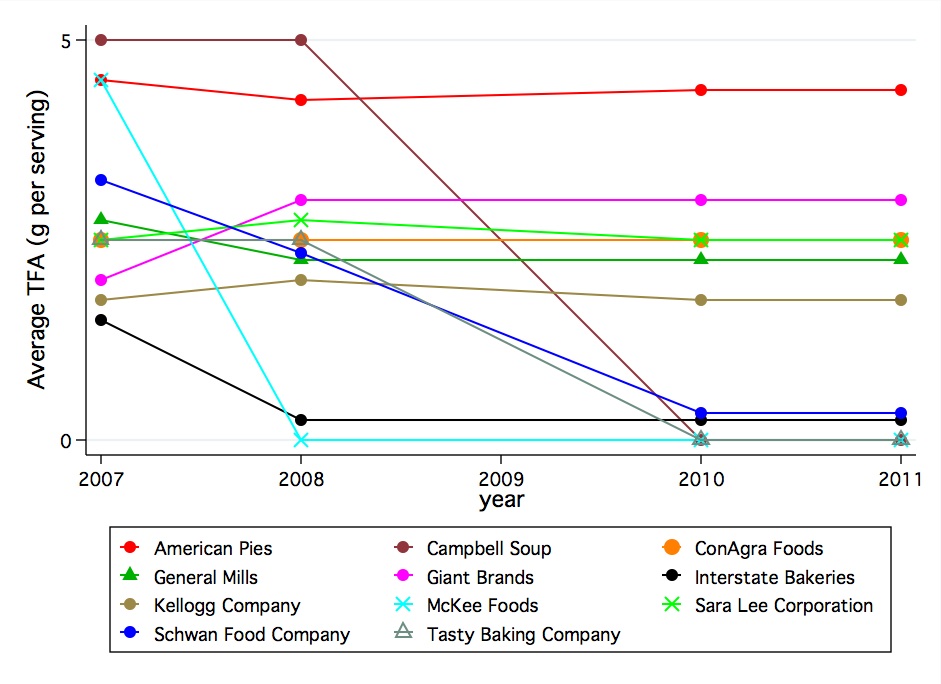


Pizzas


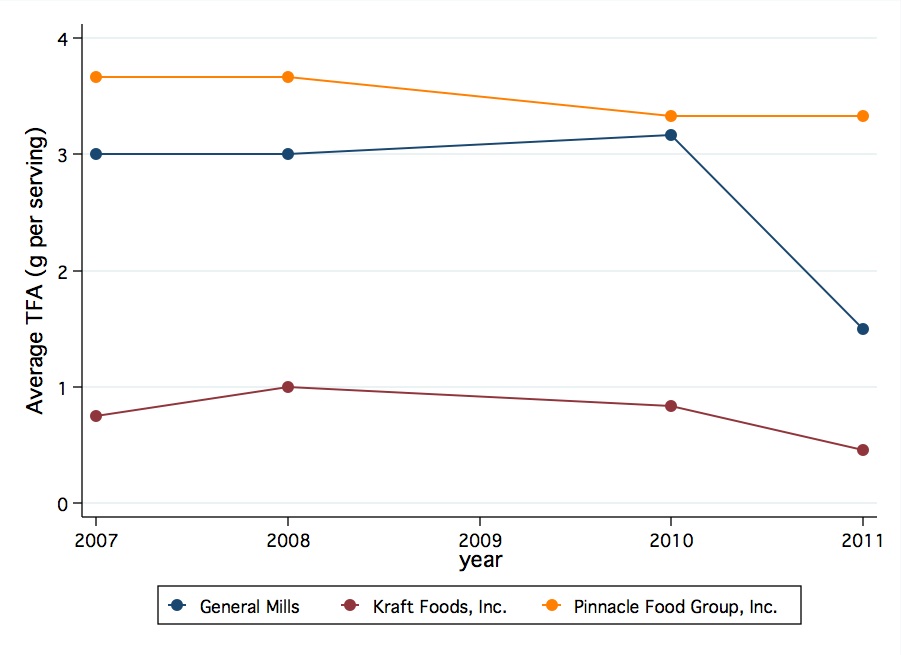


Popcorns


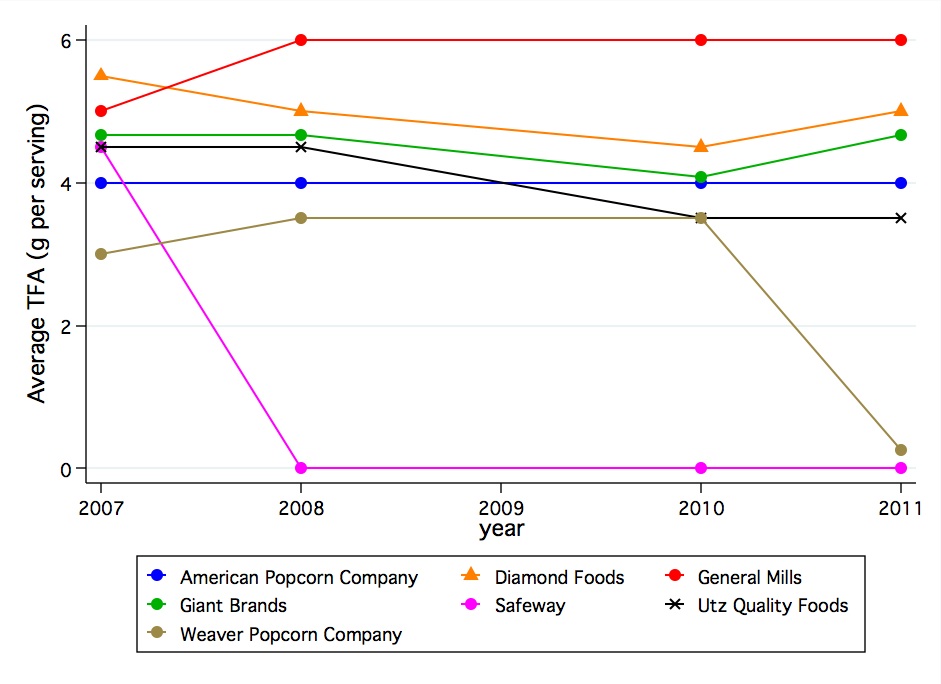


Rolls


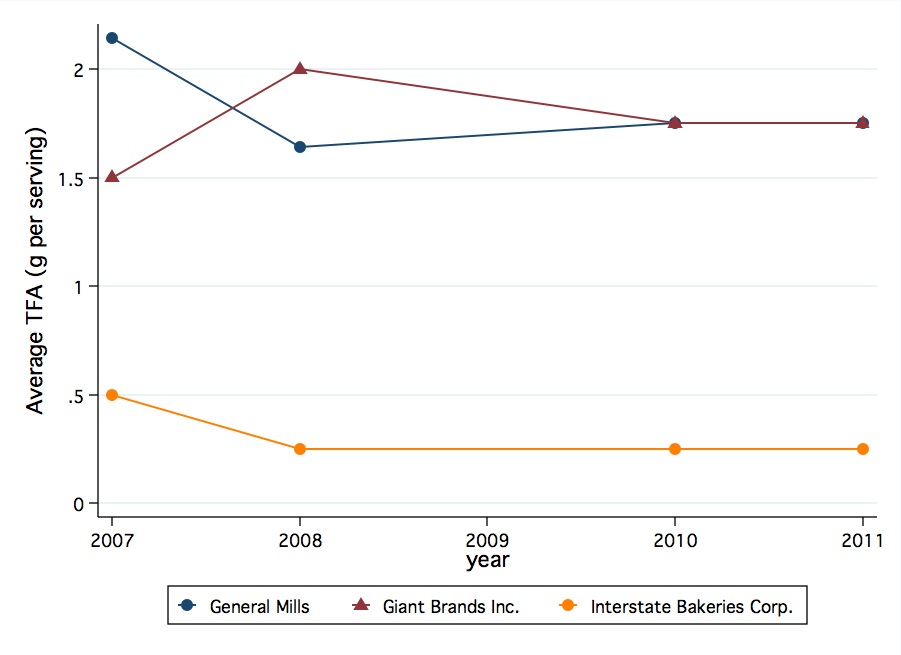

Supplement: Supplementary file 1 [file 12_0198_01.doc]
