# Supplementary material for: Trends in Trans Fatty Acids Reformulations of US Supermarket and Brand-Name Foods From 2007 Through 2011
Source: Prev Chronic Dis. 2013 May 23;10:E85. doi: 10.5888/pcd10.120198 (PMC3670643; doi:10.5888/pcd10.120198)
Supplement: Supplementary file 2 [file 12_0198_02.doc]

**Supplementary Table 1. Identified US brand-name food products that contained ≥0.5 g/serving TFA in 2007 and increased their TFA content by 2011**

| **Parent Company** | **Product** | **TFA content in 2007 (g/serving)** | **TFA content in 2011 (g/serving)** |
| --- | --- | --- | --- |
| **ConAgra Foods** | Chicken Wings (Hot and spicy variety) | 0.5 | 1.0 |
| **ConAgra Foods** | Crispy Chicken Variety Pack | 1.0 | 1.5 |
| **General Mills** | Create & Bake Cookie Dough (Chocolate Chip and Sugar varieties) | 1.5 | 2.0 |
| **General Mills** | Grands! Homestyle Biscuits | 3.0 | 3.5 |
| **General Mills** | Old Fashioned Kettle Corn Popcorn | 5.0 | 6.0 |
| **General Mills** | Ready to Bake Cookie Dough (Chocolate Chip) | 1.0 | 2.0 |
| **General Mills** | Ready to Bake Cookie Dough (Sugar Cookie) | 1.5 | 2.0 |
| **Giant Brands** | Crescent Dinner Rolls | 1.5 | 2.0 |
| **Giant Brands** | Graham Cracker Pie Crust | 2.0 | 3.0 |
| **Giant Brands** | Jumbos Buttermilk Biscuits | 2.0 | 3.0 |
| **H.J Heinz Company** | Turkey Breast Medallions | 0.5 | 1.0 |
| **On-Cor Frozen Foods** | Breaded Veal Permagiana | 0.3* | 1.0 |
| **Walmart Stores** | Stick Margarine | 2.5 | 3.0 |

*****Nutrition Facts panel listed product as containing 0.3g/serving of TFA. Product assumed to contain 0.5g/serving of TFA as food labeling regulation requires TFA content of foods be presented to the nearest 0.5g/serving.

**Supplementary Table 2. Average TFA content from 2007 to 2011 of brand-name U.S. supermarket food products that contained ≥0.5 g/serving TFA in 2007, according to food categories.**

|  |  | **TFA content in g/serving by year*** | | | |
| --- | --- | --- | --- | --- | --- |
|  |  | **2007**† | **2008**† | **2010**† | **2011**† |
| **Breads (15)** | Mean (SD) | 1.7 ± 0.7 | 0.6 ± 0.8 | 0.3 ± 0.6 | 0.5 ± 0.8 |
|  | Median (IQR )‡ | 2.0 (1.0, 2.0) | 0.3 (0.0, 1.0) | 0.0 (0.0, 0.3) | 0.0 (0.0, 1.0) |
| **Breakfasts (8)** | Mean (SD) | 1.5 ± 0.9 | 1.1 ± 0.9 | 0.8 ± 1.0 | 0.8 ± 1.0 |
|  | Median (IQR )‡ | 1.8 (0.5, 2.0) | 1.0 (0.4, 1.5) | 0.4 (0.3, 1.0) | 0.4 (0.3, 1.0) |
| **Cakes and pastries (39)** | Mean (SD) | 1.8 ± 1.3 | 1.1 ± 1.0 | 0.8 ± 0.9 | 0.7 ± 0.7 |
|  | Median (IQR )‡ | 1.5 (1.0, 2.0) | 1.0 (0.3, 1.5) | 0.3 (0.3, 1.5) | 0.3 (0.3, 1.5) |
| **Cookies, biscuits and bars (39)** | Mean (SD) | 1.9 ± 1.0 | 1.2 ± 1.1 | 1.2 ± 1.2 | 1.1 ± 1.1 |
|  | Median (IQR )‡ | 1.5 (1.0, 2.5) | 1.0 (0.3, 1.5) | 1.0 (0.3, 2.0) | 1.0 (0.0, 2.0) |
| **Crackers (15)** | Mean (SD) | 2.3 ± 1.3 | 1.4 ± 1.3 | 0.4 ± 0.5 | 0.4 ± 0.5 |
|  | Median (IQR )‡ | 2.0 (1.0, 3.5) | 1.0 (0.3, 2.0) | 0.0 (0.0, 1.0) | 0.0 (0.0, 1.0) |
| **Doughnuts (7)** | Mean (SD) | 3.1 ± 1.8 | 0.3 ± 0.0 | 0.3 ± 0.0 | 0.3 ± 0.0 |
|  | Median (IQR )‡ | 4.0 (0.5, 4.0) | 0.3 (0.3, 0.3) | 0.3 (0.3, 0.3) | 0.3 (0.3, 0.3) |
| **French fries/other potatoes (18)** | Mean (SD) | 1.3 ± 0.6 | 0.1 ± 0.3 | 0.1 ± 0.1 | 0.1 ± 0.1 |
|  | Median (IQR )‡ | 1.0 (0.5, 2.0) | 0.0 (0.0, 0.3) | 0.0 (0.0, 0.3) | 0.0 (0.0, 0.3) |
| **Ice creams (7)** | Mean (SD) | 1.4 ± 1.3 | 0.3 ± 0.3 | 0.3 ± 0.3 | 0.2 ± 0.2 |
|  | Median (IQR )‡ | 1.0 (0.5, 2.0) | 0.3 (0.0, 0.5) | 0.3 (0.0, 0.5) | 0.3 (0.0, 0.3) |
| **Margarines (11)** | Mean (SD) | 2.0 ± 0.6 | 2.0 ± 0.5 | 1.7 ± 0.9 | 1.6 ± 0.8 |
|  | Median (IQR )‡ | 2.0 (1.5, 2.5) | 2.0 (1.5, 2.5) | 2.0 (1.0, 2.5) | 1.5 (1.0, 2.5) |
| **Meats and seafood (41)** | Mean (SD) | 1.2 ± 0.9 | 0.8 ± 0.6 | 0.6 ± 0.6 | 0.6 ± 0.6 |
|  | Median (IQR )‡ | 1.0 (0.5, 1.5) | 0.5 (0.5, 1.0) | 0.5 (0.3, 1.0) | 0.5 (0.0, 1.0) |
| **Muffins (5)** | Mean (SD) | 0.7 ± 0.3 | 0.6 ± 0.5 | 0.3 ± 0.1 | 0.25 ± 0.0 |
|  | Median (IQR )‡ | 0.5 (0.5, 1.0) | 0.5 (0.3, 0.5) | 0.3 (0.3, 0.3) | 0.3 (0.3, 0.3) |
| **Pasta (10)** | Mean (SD) | 1.1 ± 0.8 | 1.2 ± 1.2 | 0.5 ± 0.6 | 0.4 ± 0.4 |
|  | Median (IQR )‡ | 0.5 (0.5, 1.5) | 0.5 (0.5, 2.0) | 0.3 (0.0, 0.5) | 0.3 (0.0, 0.5) |
| **Pies (22)** | Mean (SD) | 3.2 ± 1.1 | 2.6 ± 1.4 | 1.7 ± 1.6 | 1.7 ± 1.6 |
|  | Median (IQR )‡ | 3.0 (2.5, 4.0) | 2.5 (2.0, 3.5) | 1.8 (0.3, 3.0) | 1.8 (0.3, 3.0) |
| **Pizzas (12)** | Mean (SD) | 2.0 ± 1.6 | 2.2 ± 1.5 | 2.0 ± 1.6 | 1.4 ± 1.5 |
|  | Median (IQR )‡ | 1.3 (0.8, 3.8) | 1.5 (1.0, 3.8) | 1.3 (1.0, 3.8) | 1.0 (0.3, 2.0) |
| **Popcorns (11)** | Mean (SD) | 4.5 ± 1.3 | 4.2 ± 1.8 | 3.8 ± 2.1 | 3.8 ± 2.2 |
|  | Median (IQR )‡ | 4.5 (4.0, 6.0) | 4.5 (3.5, 6.0) | 4.5 (3.5, 6.0) | 4.0 (2.0, 6.0) |
| **Rolls (10)** | Mean (SD) | 1.9 ± 0.6 | 1.6 ± 1.0 | 1.6 ± 0.8 | 1.6 ± 0.8 |
|  | Median (IQR )‡ | 2.0 (1.5, 2.5) | 1.8 (0.3, 2.5) | 1.8 (1.5, 2.0) | 1.8 (1.5, 2.0) |

* Data were not collected in 2009.

† All products listing 0g TFA but still containing partially hydrogenated oils in the ingredients list were considered to still contain 0.25g/serving of TFA.

‡ IQR=interquartile range (25^th^, 75^th^ percentile).

**Supplementary Table 3. Average TFA content from 2007 to 2011 of 236 brand-name U.S. supermarket food products that contained ≥0.5 g/serving TFA in 2007, by their major (>3 products) parent companies**

|  |  | **TFA content in g/serving by year*** | | | |
| --- | --- | --- | --- | --- | --- |
|  |  | **2007**† | **2008**† | **2010**† | **2011**† |
| **American Pies (4)** | Mean (SD) | 4.5 ± 0.4 | 4.3 ± 0.6 | 4.4 ± 0.3 | 4.4 ± 0.3 |
|  | Median (IQR) | 4.5 (4.3, 4.8) | 4.3 (3.8, 4.8) | 4.5 (4.3, 4.5) | 4.5 (4.3, 4.5) |
| **Campbell Soup (12)** | Mean (SD) | 2.3 ± 1.6 | 2.1 ± 1.7 | 0.8 ± 1.1 | 0.8 ± 0.9 |
|  | Median (IQR )‡ | 2.0 (1.3, 3.3) | 1.5 (0.8, 3.3) | 0.1 (0.0, 1.5) | 0.3 (0.0, 1.8) |
| **Cole’s Quality Foods (4)** | Mean (SD) | 1.6 ± 0.5 | 0.0 ± 0.0 | 0.0 ± 0.0 | 0.0 ± 0.0 |
|  | Median (IQR )‡ | 1.8 (1.3, 2.0) | 0.0 (0.0, 0.0) | 0.0 (0.0, 0.0) | 0.0 (0.0, 0.0) |
| **ConAgra Foods (14)** | Mean (SD) | 1.4 ± 0.5 | 1.3 ± 0.6 | 1.1 ± 0.8 | 1.1 ± 0.7 |
|  | Median (IQR )‡ | 1.5(1.0, 1.5) | 1.3 (1.0, 1.5) | 1.3 (0.5, 1.5) | 1.3 (1.0, 1.5) |
| **Continental Mills (4)** | Mean (SD) | 1.1 ± 0.6 | 1.1 ± 0.6 | 0.6 ± 0.9 | 0.5 ± 0.7 |
|  | Median (IQR )‡ | 1.0 (0.8, 1.5) | 1.0 (0.8, 1.5) | 0.3 (0.1, 1.1) | 0.3 (0.1, 0.9) |
| **General Mills (48)** | Mean (SD) | 1.9 ± 1.0 | 1.7 ± 1.2 | 1.6 ± 1.2 | 1.4 ± 1.1 |
|  | Median (IQR )‡ | 1.8 (1.0, 2.3) | 1.5 (1.0, 2.0) | 1.5 (0.8, 2.0) | 1.5 (0.5, 2.0) |
| **Giant Brands (12)** | Mean (SD) | 2.4 ± 1.8 | 2.5 ± 1.8 | 2.2 ± 2.0 | 2.4 ± 2.0 |
|  | Median (IQR )‡ | 2.0 (1.3, 2.5) | 2.3 (1.5, 2.8) | 2.0 (0.6, 2.8) | 2.0 (0.9, 3.0) |
| **H.J. Heinz (18)** | Mean (SD) | 1.3 ± 0.6 | 0.4 ± 0.5 | 0.4 ± 0.5 | 0.5 ± 0.6 |
|  | Median (IQR )‡ | 1.0 (1.0, 2.0) | 0.3 (0.0, 0.5) | 0.3 (0.0, 0.5) | 0.3 (0.0, 1.0) |
| **Interstate Bakeries (13)** | Mean (SD) | 1.1 ± 0.6 | 0.4 ± 0.4 | 0.3 ± 0.2 | 0.2 ± 0.1 |
|  | Median (IQR )‡ | 1.0 (0.5, 1.5) | 0.3 (0.3, 0.3) | 0.3 (0.3, 0.3) | 0.3 (0.3, 0.3) |
| **Kellogg Company(22)** | Mean (SD) | 2.3 ± 1.4 | 0.9 ± 1.3 | 0.3 ± 0.5 | 0.3 ± 0.5 |
|  | Median (IQR )‡ | 2.0 (1.5, 3.0) | 0.3 (0.0, 1.5) | 0.0 (0.0, 0.3) | 0.0 (0.0, 0.3) |
| **Kraft Foods (8)** | Mean (SD) | 0.8 ± 0.3 | 0.9 ± 0.4 | 0.8 ± 0.3 | 0.5 ± 0.4 |
|  | Median (IQR )‡ | 0.8 (0.5, 1.0) | 1.0 (0.5, 1.0) | 1.0 (0.5, 1.0) | 0.4 (0.3, 1.0) |
| **Nestlé (8)** | Mean (SD) | 1.2 ± 1.2 | 0.5 ± 0.6 | 0.4 ± 0.7 | 0.2 ± 0.2 |
|  | Median (IQR )‡ | 0.8 (0.5, 1.3) | 0.5 (0.1, 0.5) | 0.3 (0.0, 0.5) | 0.1 (0.0, 0.4) |
| **PepsiCo (4)** | Mean (SD) | 1.5 ± 0.7 | 1.3 ± 0.3 | 1.5 ± 0.7 | 1.1 ± 0.3 |
|  | Median (IQR )‡ | 1.3 (1.0, 2.0) | 1.3 (1.0, 2.0) | 1.3 (1.0, 2.0) | 1.0 (1.0, 1.3) |
| **Pinnacle Food Group (6)** | Mean (SD) | 2.6 ± 1.6 | 2.6 ± 1.6 | 2.2 ± 1.7 | 2.2 ± 1.7 |
|  | Median (IQR )‡ | 2.0 (1.5, 4.0) | 1.8 (1.5, 4.0) | 1.5 (1.5, 3.5) | 1.5 (1.5, 3.5) |
| **Safeway (11)** | Mean (SD) | 1.9 ± 1.2 | 1.0 ± 1.2 | 0.8 ± 1.1 | 0.7 ± 1.2 |
|  | Median (IQR )‡ | 1.5 (1.0, 2.5) | 0.5 (0.0, 1.5) | 0.3 (0.0, 1.5) | 0.0 (0.0, 1.5) |
| **Sara Lee Corp. (9)** | Mean (SD) | 2.0 ± 0.9 | 1.6 ± 1.3 | 1.5 ± 1.3 | 1.4 ± 1.3 |
|  | Median (IQR )‡ | 1.5 (2.0, 3.0) | 1.5 (0.3, 3.0) | 2.0 (0.3, 3.0) | 1.5 (0.3, 2.5) |
| **Schwan Food Company (7)** | Mean (SD) | 2.9 ± 1.2 | 2.0 ± 1.5 | 0.3 ± 0.4 | 0.3 ± 0.4 |
|  | Median (IQR )‡ | 3.5 (2.5, 3.5) | 2.5 (0.0, 3.5) | 0.3 (0.0, 0.5) | 0.3 (0.0, 0.5) |
| **Tasty Baking Company (10)** | Mean (SD) | 2.8 ± 2.2 | 0.5 ± 0.7 | 0.2 ± 0.1 | 0.2 ± 0.1 |
|  | Median (IQR )‡ | 2.0 (1.0, 4.0) | 0.3 (0.3, 0.3) | 0.3 (0.3, 0.3) | 0.3 (0.3, 0.3) |
| **Unilever (7)** | Mean (SD) | 1.5 ± 1.0 | 1.1 ± 1.0 | 0.8 ± 0.9 | 0.7 ± 0.8 |
|  | Median (IQR )‡ | 2.0 (0.5, 2.5) | 0.5 (0.3, 2.0) | 0.3 (0.3, 2.0) | 0.3 (0.3, 1.5) |
| **Walmart Stores (5)** | Mean (SD) | 2.0 ± 0.4 | 1.2 ± 1.2 | 0.7 ± 1.3 | 0.7 ± 1.3 |
|  | Median (IQR )‡ | 2.0 (2.0, 2.0) | 1.5 (0.0, 2.0) | 0.0 (0.0, 0.3) | 0.0 (0.0, 0.3) |

* Data were not collected in 2009.

† All products listing 0g TFA but still containing partially hydrogenated oils in the ingredients list were considered to still contain 0.25g/serving of TFA.

‡ IQR=interquartile range (25^th^, 75^th^ percentile).

**Supplementary Table 4.Numbers of products originally containing ≥0.5 g/serving TFA in 2007 that were discontinued by 2011, by food category.**

| **Food Category** | **No. of Products Discontinued by 2011 / Total Products in 2007(percent)*** |
| --- | --- |
| **Breads** | 4 / 19 (21.1%) |
| **Breakfasts** | 1 / 9 (11.1%) |
| **Cakes and other pastries** | 22 / 61 (36.1%) |
| **Cookies, biscuits and bars** | 13 / 52 (25.0%) |
| **Crackers** | 2 / 17 (11.8%) |
| **Doughnuts** | 1 / 8 (12.5%) |
| **French fries/other potatoes** | 4 / 22 (18.2%) |
| **Ice creams** | 9 / 16 (56.3%) |
| **Margarines** | 1 / 12 (8.3%) |
| **Meats and seafood** | 14 / 55 (25.5) |
| **Muffins** | 0 / 5 (0.0%) |
| **Pasta** | 8 / 18 (44.4%) |
| **Pies** | 5 / 27 (18.5%) |
| **Pizzas** | 3 / 15 (20.0%) |
| **Popcorns** | 1 / 12 (8.3%) |
| **Rolls** | 2 / 12 (16.7%) |

*****Chi-square test P <0.001 comparing the different proportions of discontinued products by food category.

**Supplementary Table 5.Sensitivity analyses evaluating TFA reformulations between 2007 and 2011 among all 360 major US brand-name food products containing≥0.5 g/serving TFA in 2007, including 90 products discontinued by 2011.***

|  | **Year**† | | | |
| --- | --- | --- | --- | --- |
|  | **2007** | **2008** | **2010** | **2011** |
| **Number of products containing any TFA (% of total)** ‡ | 360 (100) | 251 (70 ) | 208 (58) | 197 (55) |
| **Mean ± SD TFA content, g/serving**‡§ | 1.9 ± 1.5^a^ | 1.1 ± 1.3^b^ | 0.8 ± 1.2^c^ | 0.7 ± 1.1^d^ |
| **Median (25th,75th percentile) TFAcontent in g/serving**‡ | 1.5 (1.0,2.5)^a^ | 0.5 (0.0, 1.5)^b^ | 0.3 (0.0, 1.0)^c^ | 0.3 (0.0, 1.0)^d^ |

*Ninety productssold in 2007 and discontinued by 2011 were included in the analysis, based on theactual TFA contents of the products in the years they were sold and then assuming reformulation to contain 0g of TFA per serving in all years after discontinuation.

†Data were not collected in 2009.

‡All products listing 0g TFA but still containing partially hydrogenated oils in the ingredients list were considered to still contain 0.25g/serving of TFA.

§ Values with different superscripts (^a,b,c,d^) are significantly different from each other (p <0.01); values with identical superscripts are not significantly different from each other.

**Supplementary Table 6.Sensitivity analyses evaluating changes in TFA contentsbetween 2007 and 2011 among all 360 major US brand-name food products containing≥0.5 g/serving TFA in 2007, including 90 products discontinued by 2011.***

|  | **Overall** | **Changes by Time Period** | | |  |
| --- | --- | --- | --- | --- | --- |
|  | **(2007 to 2011)** | **2007 to 2008** | **2008 to 2010**† | **2010 to 2011** | **P for trend**‡ |
| **No. of products** | 360 | 360 | 360 | 360 | n/a§ |
| **Mean TFA change, g/serving**||¶ | -1.2 ± 1.5 | - 0.8 ± 1.4^a^ | -0.3 ± 0.9^b^ | -0.1 ± 0.4^c^ | <0.0001 |
| **Mean TFA change, percent**||¶ | -61.4 ± 48.7 | - 42.3 ± 51.8^a^ | -17.5 ± 66.8^b^ | -0.9 ± 80.3^c^ | <0.0001 |

*Ninety productssold in 2007 and discontinued by 2011 were included in the analysis, based on the actual TFA contents of the products in the years they were sold and then assuming reformulation to contain 0g of TFA per serving in all years after discontinuation.

†A two-year period; data were not collected in 2009.

‡Determined from linear regression model with TFA change (either in g/serving or percent) modeled as dependent variable and time modeled as a continuous variable

§n/a = not applicable

||All products listing 0g TFA but still containingpartially hydrogenated oils in the ingredients list were considered to still contain 0.25g /serving of TFA.

¶Values with different superscripts (a,b,c) were significantly different from each other (p<0.01) across time periods; values with identical superscripts were not significantly different from each other.Mean absolute changes and mean percent changes are summed at the individual product-level, and are thus not mathematically identical
